# Role of Killer Immunoglobulin-Like Receptor and Ligand Matching in Donor Selection

**DOI:** 10.1155/2012/271695

**Published:** 2012-11-10

**Authors:** Meral Beksaç, Klara Dalva

**Affiliations:** ^1^Department of Hematology, Ankara University Unrelated Donor Registry and Cord Blood Bank, Ankara, Turkey; ^2^Ankara Tip Fakultesi Hematoloji Bilim Dali, Cebeci Yerleskesi, Dikimevi, 06620 Ankara, Turkey; ^3^HLA Typing Laboratories, Department of Hematology, Ankara University School of Medicine, İbni Sina Hospital, Sihhiye, 06100 Ankara, Turkey

## Abstract

Despite all efforts to improve HLA typing and immunosuppression, it is still impossible to prevent severe graft versus host disease (GVHD) which can be fatal. GVHD is not always associated with graft versus malignancy and can prevent stem cell transplantation from reaching its goals. Overall T-cell alloreactivity is not the sole mechanism modulating the immune defense. Innate immune system has its own antigens, ligands, and mediators. The bridge between HLA and natural killer (NK) cell-mediated reactions is becoming better understood in the context of stem cell transplantation. Killer immunoglobulin-like receptors (KIRs) constitute a wide range of alleles/antigens segregated independently from the HLA alleles and classified into two major haplotypes which imprints the person's ability to suppress or to amplify T-cell alloreactivity. This paper will summarize the impact of both activating and inhibitory KIRs and their ligands on stem cell transplantation outcome. The ultimate goal is to develop algorithms based on KIR profiles to select donors with maximum antileukemic and minimum antihost effects.

## 1. Introduction

 Allogeneic hematopoietic stem cell transplantation is a curative approach. Removal of residual malignant cells and relapse prevention by an intensive conditioning regimen and reinstitution of a successful posttransplant anticancer immune response are the essential benefits of this treatment modality. The current donor-recipient matching criteria involve multiple factors but the only immunological barrier taken into consideration is the human leukocyte antigen system (HLA). However, an important factor affecting the success is the function of natural killer (NK) cells which are closely controlled by KIRs that interact with specific HLA class I ligands. KIR genes are encoded within 100–200 kb region of the leukocyte receptor complex (LRC) located on chromosome 19 (19q13.4) and segregated independently from the HLA genes. Most of HLA identical donor-recipient matched pairs are actually KIR mismatched. Innate system involves natural killer cells which through binding to their ligands can inhibit or activate the anticancer or antidonor reactivity arising from HLA recognition. The KIR genes belong to the most polymorphic structures between all surface receptors, second only to MHC, and are the key regulators of NK cells. Since these receptors are located on natural killer cells, they are called killer immunoglobulin-like receptors (KIRs). KIRs may exert inhibitory or activating functions through iKIR and aKIRs. There are nine iKIR and six aKIR receptors. The number of Ig-like domains on their extracellular region and the length of the cytoplasmic tail of the KIR proteins define the acronym for each KIR gene. Most of them have 2 Ig-like domains D1, D2 or D0, D2 (KIR2D), and the others have 3 domains D0, D1, and D2 (KIR3D). Receptor families with a long tail, “L” KIRs, are mostly inhibitory (e.g., KIR2DL, KIR3DL); whereas short tail ones, “S” KIRs, are mostly activating (e.g., KIR2DS, KIR3DS) with an exception, KIR2DL4, which has a potential for activating or inhibitory function. Some but not all of natural killer receptor ligands have been defined. Some are HLA class I molecules including HLA-A (A3, A11) for KIR3DL2, HLA-B (Bw4) for KIR3DL1, HLA-C^Lys80^, C^Asn80^, for KIR2DL1 and KIR2DL2/3, respectively, and HLA-G for KIR2DL4 antigen subgroups. The HLA-C ligands are grouped according to their residue on position 80. The acronym of group 1 HLA-C is C1 or C^Lys80^ (HLA*C 01, 03, 07, 08, 12, 13, 14, and 16 and B*4601, B*7301) and group 2 HLA-C is C2 or C^Asn80^ (HLA*C 02, 04, 05, 06, 15, 17, and 18) [1, 2].

KIR diversity among people may originate from three reasons: allelic variations, the level of expression on the cell surface, and the haplotypic variability. Based on population studies KIR alleles are organized into two broad haplotypes: haplotype A and B. Haplotype A constitutes of 7 KIR genes, 6 inhibitory KIRs including the 4 framework genes plus the only activating gene KIR2DS4. Haplotype B is characterized by the presence of 1–5 activating KIR genes beside the increased number of genes with a greater variability, generated from recombinations of a centromeric and a telomeric cluster. Homozygosity of Haplotype A versus B defines an individual's ability to amplify or suppress immune reactions. Since NK cells can recognize donor antigens from tumor antigens, at least normal NK cell reactivity is essential for a graft versus leukemia effect in the absence of graft versus host reaction. However, to complicate the events further, even in the presence of activating KIR genes, these reactions can be silenced leading to abolition of activity.

Many investigators have evaluated the role of KIR receptor polymorphisms, KIR receptor-ligand matching on transplant outcome. The variation among studies in regard to donor or stem cell types, conditioning regimens, use of T-cell depletion has demonstrated a complex picture. To complicate analysis even further, factors that increase relapse or GVHD rates, such as disease activity at transplantation or gender matching, are not always similar between these studies. It is hypothesized that KIR-ligand mismatching is prerequisite for NK alloreactivity and, thus HLA mismatched transplants exert the best models for studying innate immune system activities [1, 2].

Previous reviews have grouped these NK alloreactivity studies in four models:

1. KIR-ligand incompatibility, or ligand-ligand model (Ruggeri et al.);
2. receptor-ligand model (Leung et al.);
3. KIR gene-gene (receptor-receptor or haplotype) model (initially described by Nantes group, actually is similar to the Stanford model) (Gagne et al., Parham et al., and McQueen et al.);
4. missing ligand model (retrospective model actually similar to the receptor-ligand model but neither the donor KIR nor HLA is considered for donor selection).

In summary, except for the fourth model which is a retrospective evaluation, the other models are based on biological matching principles and are being used for donor selection [1–5].

In this paper, these reports will be categorized and summarized according to stem cell source, donor matching, and conditioning regimens (Tables 1 and 2). It is an attempt towards a guide for use of KIR allele-ligand matching in donor selection.

## 2. KIR Matching in Haploidentical Stem Cell Transplants

Although NK cells activity against malignant cell were known for a long time, it was only in late 90s that the impact of KIR-ligands on allogeneic transplant was investigated. Haploidentical transplants have been an ideal model to investigate these effects. Following the initial reports by Valiante and Parham, the Perugia group and later additional groups investigated the impact of donor-ligand matching status after T cell depleted haploidentical transplantation for AML [3, 6–8]. In the presence of KIR-ligand mismatch between donor-recipient pairs, improved engraftment and a decrease in relapse rates were observed [1–3]. These effects were restricted to patients transplanted only in CR. However, subsequent studies were not able to confirm these results, directing investigators to analyze additional parameters. Recently, a study on haploidentical transplants with a posttransplant cyclophosphamide infusion have confirmed the role of haplotype B to have a GVL effect and prolongation of survival similar to the results obtained between siblings or matched unrelated subjects [7]. Multiple factors such as high T-cell content of the graft, suboptimal dose of T-cell depletion and HLA_mismatch level may effect NK-cell reconstitution and mask KIR effects [2]. A recent publication supports the following statement: T-cell alloreactivity overrides NK-mediated responses and optimal immunosuppression liberates NK-cell effects against leukemia. In other words, if extensive T-cell depletion such as CD34+ selection is performed, such as the setting of haploidentical transplantation, KIR-ligand mismatching benefits become visible [2, 3, 6–8].

## 3. KIR Gene-Gene Matching in Sibling Identical Stem Cell Transplants

As seen in Tables 1 and 2, Hsu, McQueen, Verheyden, Kim, Dalva, and Stringaris published reports analyzing the impact of KIRs utilizing KIR genotype, KIR haplotype, or telomeric KIR haplotype matching models [5, 10–14]. Four of these studies showed a beneficial effect of the B haplotype,which contains more activating KIRs, on both survival and relapse. These results are in accordance with results observed following haploidentical transplants by Ruggeri et al. [3] and Symons et al. [7]. It is important to note that both of the inconsistent studies include in vivo T depletion which might have unleashed NK-cell alloreactivities. However even the results from these two studies are not similar.

## 4. KIR Matching in Unrelated Myeloablative Stem Cell Transplants among Adults

Donor KIR3DS1, which is an activating KIR and is part of the haplotype B, is observed among 33% of donors. Transplants performed with stem cells from donors positive for 3DS1 led to a decrease in grade II-IV GVHD and TRM without increasing relapse rate. This effect was amplified among subjects who were homozygous for this phenotype [27]. These authors have also reported a similar GVHD protection effect of Bw4 that was amplified in the presence of 3DS1. The overall effect of haplotype B on GVHD was dependent on 3DS1 and the other aKIR, 2DS2. These aKIRs are in strong linkage disequilibrium. Thus it was concluded that donor KIR 3DS1 and Bw4 expression, additively protects recipients from GVHD and TRM, without hampering the GvL effect.

## 5. KIR Matching in Unrelated Cord Blood Transplants

Similar to haploidentical transplants, cord blood transplants also utilize highly mismatched donors allowing a prominent GVHD effect that can be investigated under the context of KIR matching [28, 29]. So far, there are two major reports with opposing results. As seen in Tables 1 and 2, the type of conditioning regimens, in vivo T depletion, and number of cord blood units were different between these studies leading to a detrimental effect of KIRs following reduced intensity conditioning regimen. Based on existing data, it is not possible to establish criteria for cord blood selection. There is certainly need for prospective studies analyzing the effect of KIRs and KIR-ligand matching in both GVH or HVG directions.

## 6. KIR Matching for Donor Selection

Finally, the first attempts of donor selection criteria based on KIR genotyping have been proposed: the studies by Cooley et al. demonstrated protection against relapse and survival benefit when donors with certain KIR B genotypes are used for T-cell replete unrelated donor HCT for AML suggesting KIR genotyping to be incorporated into unrelated donor selection algorithm [25, 26]. This finding is supported by data from sibling transplants, with the exception of data from Stanford and us, reporting an increase in relapse associated with haplotype B [5, 11, 12, 14]. On the contrary, Stringaris et al., also based on data from sibling transplants, have reported a positive effect of haplotype B on survival. Through three groups including us, we were able to show the presence of activating KIRs to augment graft versus host/leukemia immunity whereas the inhibitory KIRs cause immune tolerance. This effect is observed frequently, if not exclusively, among patients with myeloid disorders [11, 12, 14]. In spite of all inconsistencies and contradictory results which are usually arising from the lack of simultaneous evaluation of donor and recipient KIR status; disease or conditioning regimen type related heterogeneity between studies, Leung was able to propose a donor selection Algorithm [2] as follows.

**(I) More Than One HLA-Matched Donor Available (Sibling, Unrelated, or Cord Blood)**

 Selection of donor with receptor-ligand mismatch in KIR.
 Selection of donor with “B” haplotype in KIR.
 No need to consider KIR-ligand mismatch (as KIR-ligands always match if HLA matches).

**(II) HLA-Matched Donor Not Available; T Cells Not Depleted (Related and Unrelated)**

 Selection of donor with the least degree of HLA mismatch.
 Selection of donor with receptor-ligand mismatch in KIR.
 Selection of donor with “B” haplotype in KIR.
 Avoid donor with KIR-ligand mismatch.

**(III) HLA-Matched Donor Not Available; T Cells Depleted or Single-Unit Cord Blood Transplant**

 Selection of donor with receptor-ligand mismatch in KIR.
 Selection of donor with “B” haplotype in KIR.
 Selection of donor with KIR-ligand mismatch.

## 7. Conclusion

It appears that different KIR parameters are valid for each donor-recipient pair based on the degree of HLA matching, T-cell depletion intensity, and the type of leukemia. The protective or opposite effects of haplotype B among unrelated or sibling transplants is a perfect example for inconsistent results. Thus the algorithm presented by Leung is open to further discussion.

## Figures and Tables

**Table 1:** Characteristics of studies analyzing KIRs or KIR ligands.

Reference	*n*	Stem cell source	T-cell depletion	Donor type	HLA match	Diagnose
Ruggeri et al. 2002 [3]	92	PB	All	Related	Haploidentical	Various
Bishara et al. 2004 [6]	62	PB	Not all	Related	Haploidentical	Various
Symons et al. 2010 [7]	86	BM	None	Related	Haploidentical	Various
Weisdorf et al. 2012 [8]	24	PB	All	Related	Haploidentical	Myeloid
Cook et al. 2004 [9]	220	?	?	Related	HLA match	Lymphoid, myeloid
Hsu et al. 2005 [10]	178	BM	All	Sibling	HLA match	Various
Dalva et al. 2006 [11]	84	PB/BM	None	Sibling	HLA match	Various
McQueen et al. 2007 [5]	202	PB (89%)/BM	None	Sibling	HLA match	Various
Kim et al. 2007 [12]	53	BM/PB	None	Sibling	HLA match	Myeloid
Giebel et al. 2009 [13]	100	PB/BM	All	Sibling/unrelated	HLA match (81%)	Various
Stringaris et al. 2010 [14]	246	PB/BM	All	Sibling	HLA match	Myeloid
Davies et al. 2002 [15]	175	BM	34%	Unrelated	HLA mismatch	Various
Giebel et al. 2003 [16]	130	BM (96%)	81%	Unrelated	HLA match (47%)	Various
Bornhäuser et al. 2004 [17]	118	BM/PB	All	Unrelated	HLA match (46%)	Myeloid
Schaffer et al. 2004 [18]	190	BM/PB	All	Unrelated	HLA match (49%)	Various
Beelen et al. 2005 [19]	374	BM/PB	None	Unrelated (60%)	HLA match (63%)	CML (63 %)
De Santis et al. 2005 [20]	104	BM/PB	14% (BM)	Unrelated	HLA mismatch	Various
Kröger et al. 2005 [21]	73	PB (63%)/BM	All	Unrelated	HLA match (86%)	Myeloma
Farag et al. 2006 [22]	1571	BM	None	Unrelated	HLA match (64%)	Various
Miller et al. 2007 [23]	1770	PB/BM	None	Unrelated	HLA match	Various
Yabe et al. 2008 [24]	1489	BM	All	Unrelated	HLA match	Various
Cooley 2009 [25]	448	?	None	Unrelated	HLA match (47%)	Myeloid
Cooley et al. 2010 [26]	1086	?	None	Unrelated	HLA match (50%)	Myeloid, lymphoid
Gagne et al. 2009 [4]	264	BM	None	Unrelated	HLA match (62 %)	Various
Venstrom et al. 2010 [27]	1087	BM (97%)	19%	Unrelated	HLA match (62%)	Myeloid, lymphoid
Brunstein et al. 2009 [28]	257	CB	32%	Unrelated	HLA mismatch (92%)	Various
Willemze et al. 2009 [29]	218	CB	81%	Unrelated	HLA mismatch	Various

BM: bone marrow, PB: peripheral blood, CB: cord blood, and CML: chronic myeloid leukemia.

**Table 2:** Impact of KIR or KIR ligand matching on transplant outcome.

Reference	Overall survival	aGVHD	Graft failure	Relapse
Ruggeri et al. 2002 [3]	Better (missing KIR ligand)	Decrease (missing KIR ligand)	Decrease (missing KIR ligand)	Decrease (missing KIR ligand)
Bishara et al. 2004 [6]	Better (KIR match, GVH direction)	increase (donor aKIR)	No effect	No effect
Symons et al. 2010 [7]	Better (iKIR mm, D: haplotype B)	No effect	—	Decrease (iKIRmm; haplotype B: D/R: +/−) myeloid, lymphoid
Weisdorf et al. 2012 [8]	No effect	No effect	—	No effect (KIR increase ligand mm)
Cook et al. 2004 [9]	Unknown (haplotype A: CMV reactivation )	Unknown	—	Unknown
Hsu et al. 2005 [10]	Better (missing iKIR ligand)	No effect	—	Decrease (AML, MDS, and missing iKIR ligand)
Dalva et al. 2006 [11]	Better (aKIR m)	Decrease (iKIR m)	—	Decrease (aKIR m) Increase (D: haplotype B)
McQueen et al. 2007 [5]	Worse (donor but not recipient has haplotype B)	Increase (donor but not recipient has haplotype B, also Bw4)	—	Increase (haplotype B D/R: +/−) Decrease (D: 3DL1/3DL2; R: A3/11or Bw4+)
Kim et al. 2007 [12]	Better (D: aKIR)	Increase (D: aKIR: 2DS2-4)	—	Decrease (D: aKIR)
Giebel et al. 2009 [13]	Decrease (aKIR mm and group C2+)	Increase (aKIR mm)	—	Increase (aKIR mm)
Stringaris et al. 2010 [14]	Better (D: haplotype B)	Unknown	—	Decrease (D: aKIR or haplotype B) AML
Davies et al. 2002 [15]	Worse (missing KIR ligand) myeloid	No effect	No effect	No effect
Giebel et al. 2003 [16]	Better (KIR ligand mm)	No effect	Increase (KIR ligand match)	Decrease (KIR ligand mm) myeloid
Bornhäuser et al. 2004 [17]	No effect	No effect	—	Increase (KIR ligand mm)
Schaffer et al. 2004 [18]	Worse (increase infections)	No effect	—	No effect
Beelen et al. 2005 [19]	No effect	No effect	Increase (KIR ligand mm)	Decrease (KIR ligand mm)
De Santis et al. 2005 [20]	Worse (KIR epitope mm)	Increase (NK epitope mm)	Worse (NK epitope mm)	—
Kröger et al. 2005 [21]	No effect	Not significant	—	Decrease (KIR ligand mm)
Farag et al. 2006 [22]	No effect	No effect	No effect	No effect
Miller et al. 2007 [23]	—	Increase (KIR ligand mm)	—	Decrease (both KIR ligand and HLA mm)
Yabeet al. 2008 [24]	Worse (KIR ligand mm)	Increase (KIR ligand mm; D:2DS2 )	—	No effect
Cooley et al. 2009 and 2010 [25, 26]	Better (D: haplotype B)	No effect	No effect	Decrease (D: haplotype B) AML but not ALL
Gagne 2009 [4]	No effect (D: haplotype B); Decrease (HLA identical, KIR3DL1: D+R− D: KIR3DL1+/3DS1+ R: Bw4+ R: C1 ligand−)	Increase (HLA I: 2DL5 mm HLA nonI: 2DS1mm) Decrease (HLA I: 2DS3 mm, D: haplotype B)	—	No effect (D: haplotype B) Increase (D: 3DL1+/3DS1+ R: Bw4−) Decrease (D: 3DL1+/3DS1+ R: Bw4+)
Venstrom et al. 2010 [27]	Better (D: KIR3DS1)	Decrease (D: KIR3DS1)	—	No effect (D: 3DS1)
Brunstein et al. 2009 [28]	Worse (only with RIC)	Increase (KIR ligand mm)(RIC)	—	Decrease (KIR ligand mm) (RIC)
Willemze et al. 2009 [29]	Better (KIR ligand mm)	Decrease	—	Decrease (KIR ligand mm)

M: match, mm: mismatch, RIC: reduced intensity conditioning, D/R: donor/recipient, HLA I: HLA identical, and HLA nonI: HLA nonidentical.

## References

[1] Parham P (2005). MHC class I molecules and KIRS in human history, health and survival. *Nature Reviews Immunology*.

[2] Leung W (2011). Use of NK cell activity in cure by transplant. *British Journal of Haematology*.

[3] Ruggeri L, Capanni M, Urbani E (2002). Effectiveness of donor natural killer cell aloreactivity in mismatched hematopoietic transplants. *Science*.

[4] Gagne K, Bussson M, Bignon JD (2009). Donor KIR3DL1/3DS1 gene and recipient Bw4 KIR ligand as prognostic markers for outcome in unrelated hematopoietic stem cell transplantation. *Biology of Blood and Marrow Transplantation*.

[5] McQueen KL, Dorighi KM, Guethlein LA, Wong R, Sanjanwala B, Parham P (2007). Donor-recipient combinations of group A and B KIR haplotypes and HLA class I ligand affect the outcome of HLA-matched, sibling donor hematopoietic cell transplantation. *Human Immunology*.

[6] Bishara A, de Santis D, Witt CC (2004). The beneficial role of inhibitory KIR genes of HLA class I NK epitopes in haploidentically mismatched stem cell allografts may be masked by residual donor-alloreactive T cells causing GVHD. *Tissue Antigens*.

[7] Symons HJ, Leffell MS, Rossiter ND, Zahurak M, Jones RJ, Fuchs EJ (2010). Improved survival with inhibitory killer immunoglobulin receptor (KIR) gene mismatches and KIR haplotype B donors after nonmyeloablative, HLA-haploidentical bone marrow transplantation. *Biology of Blood and Marrow Transplantation*.

[8] Weisdorf D, Cooley S, Devine S (2012). T cell-depleted partial matched unrelated donor transplant for advanced myeloid malignancy: KIR ligand mismatch and outcome. *Biology of Blood and Marrow Transplantation*.

[9] Cook M, Briggs D, Craddock C (2006). Donor KIR genotype has a major influence on the rate of cytomegalovirus reactivation following T-cell replete stem cell transplantation. *Blood*.

[10] Hsu KC, Keever-Taylor CA, Wilton A (2005). Improved outcome in HLA-identical sibling hematopoietic stem-cell transplantation for acute myelogenous leukemia predicted by KIR and HLA genotypes. *Blood*.

[11] Dalva K, Gungor F, Soydan Akcaglayan E, Beksac M (2006). Two independent effects of immunoglobulin-like receptor (KIR) allele matching between siblings: inhibitory KIR, (IKIR) mismatches are associated with graft versus host disease (GVHD) while activatory KIR matches, (AKIR) and CGVHD are associated with graft versus leukemia (GVL). *Blood*.

[12] Kim HJ, Choi Y, Min WS (2007). The activating killer cell immunoglobulin-like receptors as important determinants of acute graft-versus host disease in hematopoietic stem cell transplantation for acute myelogenous leukemia. *Transplantation*.

[13] Giebel S, Nowak I, Dziaczkowska J (2009). Activating killer immunoglobulin-like receptor incompatibilities enhance graft-versus-host disease and affect survival after allogeneic hematopoietic stem cell transplantation. *European Journal of Haematology*.

[14] Stringaris K, Adams S, Uribe M (2010). Donor KIR genes 2DL5A, 2DS1 and 3DS1 are associated with a reduced rate of leukemia relapse after HLA-identical sibling stem cell transplantation for acute myeloid leukemia but not other hematologic malignancies. *Biology of Blood and Marrow Transplantation*.

[15] Davies SM, Ruggieri L, DeFor T (2002). Evaluation of KIR ligand incompatibility in mismatched unrelated donor hematopoietic transplants. *Blood*.

[16] Giebel S, Locatelli F, Lamparelli T (2003). Survival advantage with KIR ligand incompatibility in hematopoietic stem cell transplantation from unrelated donors. *Blood*.

[17] Bornhäuser M, Schwerdtfeger R, Martin H, Frank KH, Theuser C, Ehninger G (2004). Role of KIR ligand incompatibility in hematopoietic stem cell transplantation using unrelated donors. *Blood*.

[18] Schaffer M, Malmberg KJ, Ringdén O, Ljunggren HG, Remberger M (2004). Increased infection-related mortality in KIR-ligand-mismatched unrelated allogeneic hematopoietic stem-cell transplantation. *Transplantation*.

[19] Beelen DW, Ottinger HD, Ferencik S (2005). Genotypic inhibitory killer immunoglobulin-like receptor ligand incompatibility enhances the long-term antileukemic effect of unmodified allogeneic hematopoietic stem cell transplantation in patients with myeloid leukemias. *Blood*.

[20] de Santis D, Bishara A, Witt CS (2005). Natural killer cell HLA-C epitopes and killer cell immunoglobulin-like receptors both influence outcome of mismatched unrelated donor bone marrow transplants. *Tissue Antigens*.

[21] Kröger N, Shaw B, Iacobelli S (2005). Comparison between antithymocyte globulin and alemtuzumab and the possible impact of KIR-ligand mismatch after dose-reduced conditioning and unrelated stem cell transplantation in patients with multiple myeloma. *British Journal of Haematology*.

[22] Farag SS, Bacigalupo A, Eapen M (2006). The effect of KIR ligand incompatibility on the outcome of unrelated donor transplantation: a report from the center for international blood and marrow transplant research, the European blood and marrow transplant registry, and the Dutch registry. *Biology of Blood and Marrow Transplantation*.

[23] Miller JS, Cooley S, Parham P (2007). Missing KIR ligands are associated with less relapse and increased graft-versus-host disease (GVHD) following unrelated donor allogeneic HCT. *Blood*.

[24] Yabe T, Matsuo K, Hirayasu K (2008). Donor killer immunoglobulin-like receptor (KIR) genotype-patient cognate KIR ligand combination and antithymocyte globulin preadministration are critical factors in outcome of HLA-C-KIR ligand-mismatched T cell-replete unrelated bone marrow transplantation. *Biology of Blood and Marrow Transplantation*.

[25] Cooley S, Trachtenberg E, Bergemann TL (2009). Donors with group B KIR haplotypes improve relapse-free survival after unrelated hematopoietic cell transplantation for acute myelogenous leukemia. *Blood*.

[26] Cooley S, Weisdorf DJ, Guethlein LA (2010). Donor selection for natural killer cell receptor genes leads to superior survival after unrelated transplantation for acute myelogenous leukemia. *Blood*.

[27] Venstrom JM, Gooley TA, Spellman S (2010). Donor activating KIR3DS1 is associated with decreased acute GVHD in unrelated allogeneic hematopoietic stem cell transplantation. *Blood*.

[28] Brunstein CG, Wagner JE, Weisdorf DJ (2009). Negative effect of KIR alloreactivity in recipients of umbilical cord blood transplant depends on transplantation conditioning intensity. *Blood*.

[29] Willemze R, Rodrigues CA, Labopin M (2009). KIR-ligand incompatibility in the graft-versus-host direction improves outcomes after umbilical cord blood transplantation for acute leukemia. *Leukemia*.

